# A patient with complete IFN-γR1 deficiency and tuberculosis resembling BCG disease

**DOI:** 10.70962/jhi.20250013

**Published:** 2025-06-13

**Authors:** Sara Espinosa-Padilla, Héctor Gómez-Tello, Carlos Sanchez-Flores, Uriel Pérez-Blanco, Tiareth Cova-Guzmán, Stéphanie Boisson-Dupuis, Jacinta Bustamante, Lizbeth Blancas-Galicia

**Affiliations:** 1 Laboratory of Immunodeficiency, National Institute of Pediatrics, Mexico City, Mexico; 2 Immunology Department, Hospital del Niño Poblano, Puebla, Mexico; 3 St Giles Laboratory of Human Genetics of Infectious Diseases, Rockefeller Branch, Rockefeller University, New York, NY, USA; 4 Laboratory of Human Genetics of Infectious Diseases, Necker Branch, Necker Hospital for Sick Children, Paris, France; 5 Paris Cité University, Imagine Institute, Paris, France; 6 Study Center for Primary Immunodeficiencies, Necker Hospital for Sick Children, AP-HP, Paris, France

## Abstract

A homozygous deleterious IFN-γR1 variant (c.672G>A) was identified in a child with *Mycobacterium tuberculosis* infection and erythroderma. This case demonstrates that complete autosomal recessive IFN-γR1 deficiency can present with tuberculosis resembling BCG disease in endemic regions.

## Teaser

We present a case of a patient with a deleterious variant in IFNGR1 who contracted *Mycobacterium tuberculosis*. However, the patient presented with clinical manifestations suggestive of BCG infection. It is crucial to correctly identify the type of *M. tuberculosis* to ensure effective treatment.

Autosomal recessive (AR) complete interferon-γ receptor 1 (IFN-γR1) deficiency is classified among other inborn errors of immunity (IEI), such as Mendelian susceptibility to mycobacterial diseases (MSMD) (1). These patients lack a response to IFN-γ (2, 3), and their manifestations include severe infections caused by environmental mycobacteria and *Mycobacterium bovis bacillus* Calmette–Guérin (BCG) vaccines. However, they remain susceptible to other intracellular pathogens (1, 2). AR complete IFN-γR1 deficiency is considered the most severe form of MSMD, typically manifesting before the age of 3, alongside AR complete IFN-γR2, IRF1, IFN-γ, and STAT1 deficiencies (1). In this study, we present a case of a patient with a deleterious variant, c.672G>A, in *IFNGR1* (resulting in complete deficiency), who contracted *Mycobacterium tuberculosis* infection in a high-prevalence tuberculosis region. However, the patient presented with clinical manifestations suggestive of BCG infection. The case highlights the need for microbiological methods capable of detecting specific pathogens, as the treatment for *M. tuberculosis* differs from that for BCG (specifically, pyrazinamide resistance).

**Figure 1.**
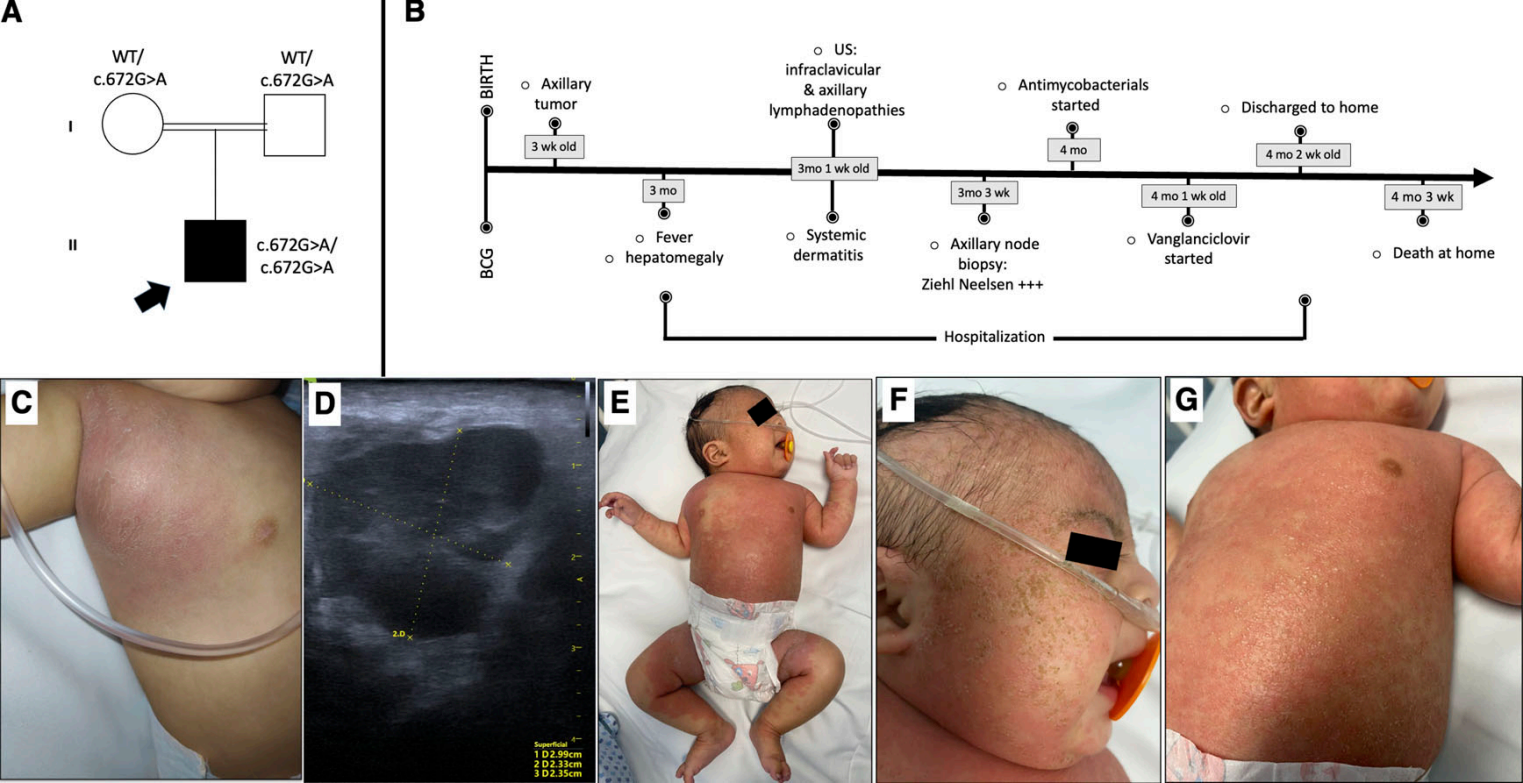
**Clinical and paraclinical findings in the described patient. (A)** Family pedigree; the patient is indicated by a black square. The presence of double lines between pedigree symbols signifies the occurrence of consanguinity. **(B)** Clinical evolution timeline. **(C)** The presence of a deformant mass is observed in the right axilla and the upper thorax. This mass shows characteristics consistent with a BCG infection. **(D)** In the ultrasound of the right axilla, a lymph node is observed with an increased size of 2.99 × 2.33 × 2.35 cm. **(E–G)** Seborrheic dermatitis on the head, diaper dermatitis in the genital area, and erythroderma on the trunk and extremities.

The 4-mo-old patient was an only child of consanguineous parents from a rural community in central Mexico (Fig. 1 A). He was born at term with an appropriate weight (3,500 g) and length (51 cm) and had no similarly affected relatives. He received a BCG vaccination (Tokyo 172 strain) in the right deltoid region at birth but developed a 20 × 20-mm mass in the right axillary region 3 wk later. Despite receiving anti-inflammatory medications, the mass did not regress. At 3 mo of age, the patient presented with tumor enlargement and fever and was admitted to the children’s hospital for evaluation. Physical examination revealed a tumor in the right axilla with associated edema and erythema in the anterior right upper thorax, and hepatomegaly was noted (Fig. 1, B and C). The clinical presentation suggested BCG infection, prompting further diagnostic investigations. Initial laboratories showed hemoglobin 7.3 g/dl (normal range [NV]: 11.0–11.2), leukocytes 38.5 × 10^9^/L (NV: 5–17.5), neutrophils 20 × 10^9^/L (NV: 1–8.5), lymphocytes 8.1 × 10^9^/L (NV: 4–13.5), eosinophils 9.6 × 10^9^/L (NV: 0.3), and platelets 3.3 × 10^9^/L (NV: 1.5–3.5). C-reactive protein levels were elevated at 96 mg/liter (NV: <7). The ultrasound revealed multiple axillary and right infraclavicular lymphadenopathies (Fig. 1 D). Biopsy of the right axillary node confirmed granulomatous lymphadenitis with numerous acid-fast bacilli seen on Ziehl–Neelsen staining and bright-field microscopy. Subsequent Xpert *Mycobacterium tuberculosis* complex/resistance to rifampin (MTB/RIF) testing confirmed *M. tuberculosis* complex infection without rifampicin resistance. The positive MTP64 antigen detected in the isolated mycobacteria from the lymph node specimen facilitated differentiation between *M. tuberculosis* and *M. bovis*. Unfortunately, imaging studies were not accessible to evaluate the organs affected by tuberculosis. The diagnosis of disseminated tuberculosis was made on the basis of clinical and paraclinical findings, and the patient initiated oral antituberculosis treatment with rifampicin (75 mg), isoniazid (50 mg), pyrazinamide (150 mg), and ethambutol (100 mg) administered every 24 h.

At the same time, the patient exhibited seborrheic dermatitis on the head, diaper dermatitis in the genital area, and erythroderma on the trunk and extremities (Fig. 1, E–G). Erythrodermic skin biopsy revealed subcorneal neutrophilic pustules, spongiosis, and perivascular lymphocytic infiltration. Toxoplasmosis, others (syphilis, hepatitis B), rubella, cytomegalovirus, herpes simplex (TORCH) screen showed positive citomegalovirus, IgM 5.76 index (NV: <0.85), IgG > 250 AU/ml (NV: <12.0), and PCR 102 IU/ml (NV: 0.0). The patient received oral valganciclovir at 16 mg/kg every 12 h for 6 wk.

Due to concerns regarding secondary immunodeficiency or congenital immunodeficiency disorder, several laboratory tests were conducted, yielding the following results: HIV antigen and antibody tests were negative; lymphocyte subpopulations results were as follows: CD3^+^ 6.2 × 10^9^/L (NV: 2.3–6.5), CD3^+^CD4^+^ 3.8 × 10^9^/L (NV: 1.5–5), CD3^+^CD8^+^ 2.0 × 10^9^/L (NV: 0.5–1.6), CD19^+^ 1.7 × 10^9^/L (NV: 0.6–3), CD16^+^CD56^+^ 4.6 × 10^9^/L (NV: 0.1–1.3); serum immunoglobulin levels were higher than the normal values: IgG 2262 mg/dl (NV: 160–570), IgA 71.6 mg/dl (NV: 5–50), IgM 439.2 mg/dl (NV: 30–100), and IgE 228 IU/dl (NV: 90); the dihydrorhodamine test for reactive oxygen species production was normal. Finally, the IEI genetic panel test revealed a predicted homozygous deleterious variant in *IFNGR1*, c.672G>A (p.Trp224*), previously associated with AR complete IFN-γR1 deficiency (2). Both parents were heterozygous carriers of this variant (c.672G>A/WT). The patient was discharged in stable condition with supervised tuberculosis treatment. In Mexico, each dose of outpatient tuberculosis treatment is supervised at home by healthcare personnel (4). Unfortunately, the parents did not bring the patient to scheduled medical appointments. Upon contacting the family, it was learned that the patient passed away at home at the age of 4 mo and 24 days; he exhibited sudden respiratory distress, consciousness, and death. As of now, there is no information on whether the patient had close contact with individuals diagnosed with tuberculosis; the parents declined epidemiological screening for tuberculosis.

Thus, we present the case of a patient with *M. tuberculosis* infection and AR complete IFN-γR1 deficiency. The causative variant c.672G>A has been previously reported in two other Amerindian children with *Mycobacterium avium*-intracellulare complex infections: one at 2 years (2) and the other at 6 years of age (3). The underlying reasons for the reported IFN-γR1–deficient patient receiving BCG and not experiencing any adverse reactions could be that the administered vaccine was suboptimal. This would preclude a definitive conclusion on the patient’s BCG immunocompetence. The patient was originally from Mexico (Puebla), a country with a high tuberculosis incidence (23 cases per 100,000 inhabitants) (4). Annually, Mexico reports 28,000 tuberculosis cases, 1% of which occur in children under 1 year of age (4). Approximately 30% of Mexican tuberculosis cases are latent (4). The patient in this study came from a low socioeconomic background, suggesting possible latent tuberculosis in a family member. The combination of mycobacterial exposure and genetic predisposition likely contributed to severe infections. This case underscores previous findings that the type of infecting microorganism in patients with MSMD and other IEI depends on their environmental exposure (5).

In patients from countries with a high prevalence of tuberculosis and BCG administration at birth, it is necessary to differentiate between BCG infection and tuberculosis; to the best of our knowledge, co-infection with BCG and tuberculosis has not been reported. MPT64 is a soluble protein encoded by a gene located in region of difference. It is expressed in *M. tuberculosis* isolates but not in the live attenuated *M. bovis* vaccine strain. The patient’s clinical course suggested BCG infection. However, the detection of MPT64 in the right axillary lymph node tissue defined *M. tuberculosis*. This case also emphasizes the need for microbiological methods capable of detecting specific pathogens, as the treatment for *M. tuberculosis* differs from that for BCG (specifically, pyrazinamide resistance) (5). This patient represents one of the earliest reported cases of AR complete IFN-γR1 deficiency. Early clinical suspicion and access to diagnostic tools were critical in identifying the disease. Unfortunately, patients with AR complete IFN-γR1 deficiency and tuberculosis may succumb to infections before diagnosis, particularly in regions with high tuberculosis incidence lacking infrastructure for timely detection of both conditions. The present paper delineates the case of an autosomal complete recessive IFN-γR1 deficiency presented with *M. tuberculosis* infection, which clinically resembled BCG infection. However, the simultaneous occurrence of BCG infection and *M. tuberculosis* infection cannot be ruled out.

## Ethics approval

This is an observational study. The Research Ethics & Committee has confirmed that no ethical approval is required.

## Consent to participate

Written informed consent was obtained from the parents.

## Consent to publish

The authors affirm the parents of the patient provided informed consent for publication of the images in Fig. 1.

## Data Availability

No/not applicable (this manuscript does not report data generation or analysis).
